# Shift in bacterioplankton diversity and structure: Influence of anthropogenic disturbances along the Yarlung Tsangpo River on the Tibetan Plateau, China

**DOI:** 10.1038/s41598-017-12893-4

**Published:** 2017-10-02

**Authors:** Peifang Wang, Xun Wang, Chao Wang, Lingzhan Miao, Jun Hou, Qiusheng Yuan

**Affiliations:** Key Laboratory of Integrated Regulation and Resources Development on Shallow Lakes, Ministry of Education, College of Environment, Hohai University, Nanjing, 210098 China

## Abstract

River systems have critical roles in the natural water environment and the transportation of nutrients. Anthropogenic activities, including wastewater discharge and river damming, raise adverse impacts on ecosystem and continuum of rivers. An increasing amount of attention has been paid to riverine bacterioplankton as they make vital contributions to biogeochemical nutrient cycle. A comprehensive study was conducted on the bacterioplankton community along the Yarlung Tsangpo River, which is the longest plateau river in China and is suffering from various anthropogenic impacts. The results indicated that nutrient variations corresponded to anthropogenic activities, and silica, nitrogen and phosphorus were retained by the dam. River damming influenced the biomass and diversity of the bacterioplankton, but significant alterations in the community structure were not observed between upstream and downstream of the dam. Moreover, the spatial distribution of the bacterioplankton community changed gradually along the river, and the dominant bacterioplankton in the upstream, midstream and downstream portions of the river were *Firmicutes*, *Bacteroidetes and Proteobacteria*, respectively. Soluble reactive phosphorus, elevation, ammonium nitrogen, velocity and turbidity were the main environmental factors that shape the bacterioplankton community. Our study offers the first insights into the variation of a bacterioplankton community of a large river in plateau region.

## Introduction

The Tibetan Plateau of China exhibits distinct energy and hydrological cycles compared with other areas. The glacier-fed rivers on the Tibetan Plateau, including the Yarlung Tsangpo River, are considered the Water Tower of Asia^1^. Apart from critical social and ecological roles in southwest China, the rivers also vitally contributed to the biogeochemical nutrient cycle^2,3^. Moreover, the anthropogenic activities have increased along the rivers on the Tibetan Plateau and the extensive hydropower potentials of the large rivers have raised additional attention^4^ since dam construction is in high demand. River damming can have significant global influences on natural water resources and river continuum, as the impoundment by dams can affect the physical, chemical and biological aspects of the water environment^5,6^.

Bacterioplankton are essential members of river ecosystems because of their important roles in the nutrient cycle and the food chain^7,8^. According to a previous study, bacterioplankton can assimilate and re-mineralize inorganic nutrients and serve as a food source for consumers^9^. In addition, they are also considered substantial contributors to the flow of energy^10–13^. Thus, the study of bacterioplankton is necessary to predict and experimentally determine their functions in the nutrient cycle in river ecosystems^8,14^. Moreover, they are diverse and dynamic as they can be influenced by different environmental factors resulting from anthropogenic disturbances^10,15–17^. While the importance of bacterioplankton diversity to the ecosystem and the threats that anthropogenic activities pose to diversity are key focuses of research in all aquatic ecosystems, river microbial diversity has received less attention than marine and lake microbial diversity^18^. In the Thames River basin, the dominant bacteria shifted from *Bacteroidetes* upstream to *Actinobacteria* downstream, and this change was driven by the sampling location^19^ rather than the physical status of the river. Similarly, in the Jiulong River Watershed, the nutrient concentrations were found to determine the bacterioplankton communities^20^. So, it is speculated that the riverine bacterioplankton community structure varied by location and nutrient concentrations^21–24^. However, other studies have demonstrated the alteration of the bacterioplankton community structure in response to physicochemical variables. The bacterioplankton communities in the Illinois River were found to be shaped by water temperature^20^, and the microbial communities in surface water of the Santa Ana River were found to be influenced by pH^25^. A more profound investigation of the relationship between bacterioplankton communities and environmental variations caused by humans is imperative for river ecosystems since there is currently no agreement. Fortunately, the recent development of culture-independent high-throughput sequencing technology has contributed to the deeper understanding of microbial variations, the factors affecting their spatial dynamics in the river, and the potential biological and biogeochemical consequences of river regulation^26,27^.

In recent decades, the Yarlung Tsangpo River basin has experienced an increase in anthropogenic activities such as urbanization and tourism. In 2015, the first water conservancy project in the main stream has been in operation, which may result in greater disturbance to the river as the river continuum and the hydrological conditions would be changed. In consequence, more attention has been paid to the effect of discharge by anthropogenic activities on water quality of great rivers in southwest China, and the alteration of biogeochemical nutrient distribution by construction of hydropower stations. However, the existing researches focused on the nutrient and sediment distribution along the mainstream of the river^28–30^. In fact, the bacterioplankton in river ecosystems also contribute to the biogeochemical nutrient cycling and can be easily affected by environmental variations^31,32^. But few studies about the bacterioplankton community variation in the Yarlung Tsangpo River were available so far. Furthermore, since several more dams have been planned to be constructed in the Yarlung Tsangpo River, it is urgent to obtain the original information to investigate the effects of anthropogenic disturbances, including river damming, on the migration of biogeochemical nutrients and the variation of bacterioplankton community in this river, providing protection and minimizing the future adverse impact on river ecosystems. Moreover, as there is only one dam in the main stream of the Yarlung Tsangpo River, the comparison between the Yarlung Tsangpo River and the highly dam-regulated rivers, such as the Lancang River (upstream of the Mekong River) and the Jinsha River (upstream of the Yangtze River) can contribute to the characterization of cascade dam effect on river bacterioplankton community, providing scientific support for the dam planning in the future.

In our study, the spatial variability of physicochemical parameters was measured. Next-generation 16 S rRNA gene sequencing combined with various multivariate statistical methods (canonical correspondence analysis and multivariate regression tree analysis) was applied to investigate the bacterioplankton community at 14 different sampling sites along the main stream of the Yarlung Tsangpo River. Temporal changes were not considered so that an intensive longitudinal sampling effort could be achieved. The main objectives of our study were to: i) taxonomically describe the distribution patterns of the bacterioplankton diversity and community structure in the Yarlung Tsangpo River, ii) determine the effects of anthropogenic disturbances on the spatial patterns of the bacterioplankton community in the river, and iii) uncover the critical environmental variables shaping the bacterioplankton community structure in the Yarlung Tsangpo River.

## Methods

### Study area and sampling procedures

**Figure 1:**
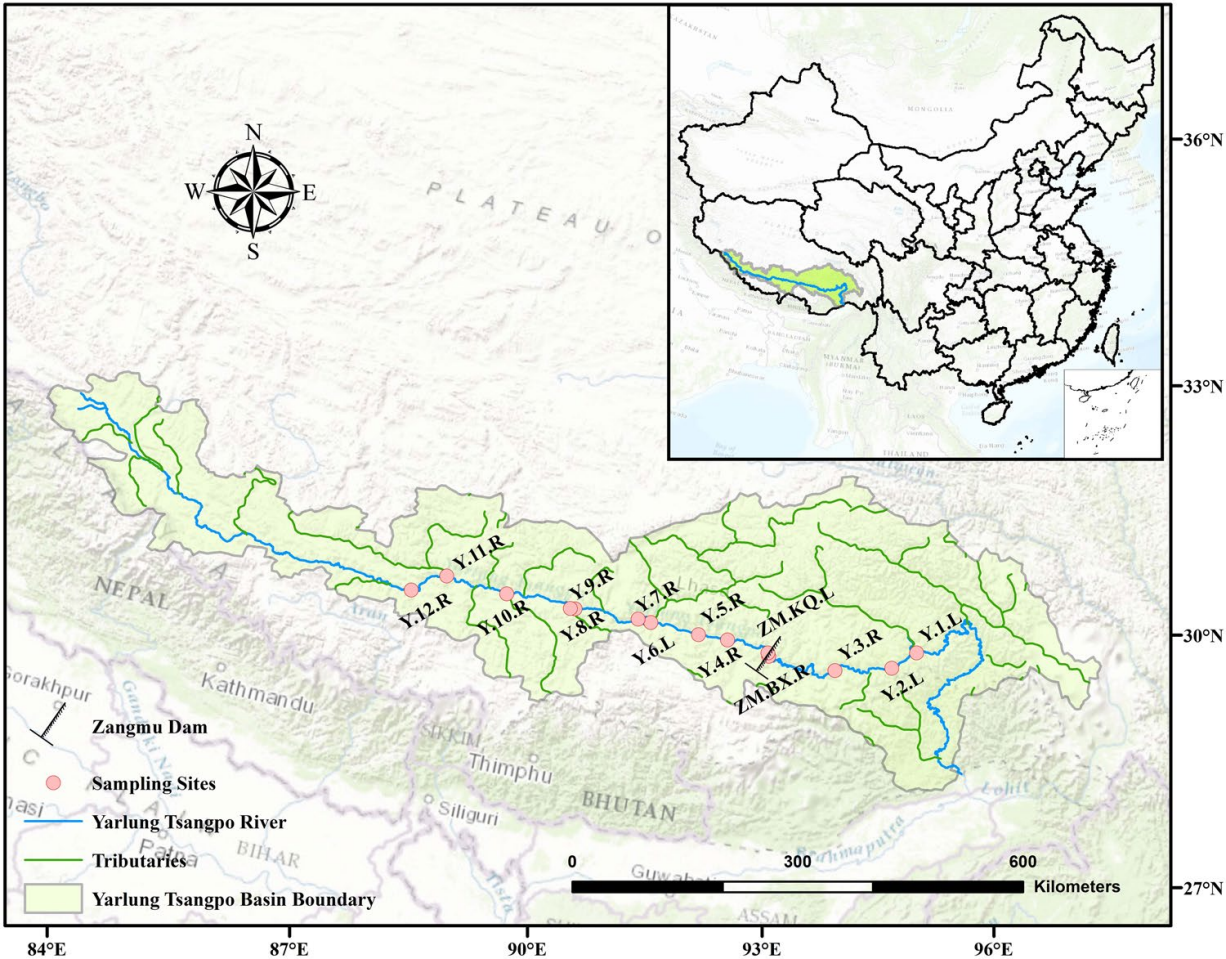
Overview and detailed map of the Yarlung Tsangpo Basin with sampling sites. Red circles present the sampling sites of our study. Blue lines and green lines indicate main stream and tributaries of the Yarlung Tsangpo River, respectively. The map was created using ArcGIS (version 10.1, Environmental Systems Research Institute, Redlands, CA).

The Yarlung Tsangpo River is located on the Tibetan Plateau and originates from the northern slope of the Himalaya Mountain^30^. It is the longest plateau river in China with a total length of 2840 km and a catchment area of 9.35 × 10^5^ km^2^. The average elevation of the river is more than 4200 m^33^. The midstream brings together many tributaries and floodplain, and there are developed terraces. Downstream, the Zangmu Dam, which is the first dam in the main stream of the Yarlung Tsangpo River with a reservoir capacity of 8.66 × 10^7^ m^3^, was constructed and began operation in 2015. Sampling was conducted along the mainstream of the Yarlung Tsangpo River from Pai County to Lhazê County in November 2016 (Fig. 1). Fourteen sampling sites extended over a length of 940 km and an elevation gradient from 2925 to 4005 m. The latitudes, longitudes and elevations of the 14 sites were determined by a portable global positioning system (GPSMAP 62 s, Garmin, KS, USA), and the details are shown in Table S1 in the Supplementary Information. Water samples were collected using a Ruttner sampler (Hydro-Bios, Altenholz, Germany) at a water depth of approximately 0.5 m. At each sampling site, three water samples were taken and were then homogenized as one sample. A 1 L water sample from each site was collected for subsequent physicochemical analysis in the laboratory. A total of 2 L of river water was filtered through 0.22 μm pore-sized prewashed polycarbonate filters (47 mm diameter, Millipore, MA, USA) by vacuum filtration to collect the microorganisms. The filters were immediately stored at −20 °C in the field and subsequently stored at −80 °C in the laboratory prior to deoxyribonucleic acid (DNA) extraction.

### Physicochemical analysis

The temperature, pH, electrical conductivity (EC), dissolved oxygen (DO) and oxidation-reduction potential (ORP) were measured *in situ* during sampling using a multiparameter water quality analyser (HQ40d, Hach, CO, USA). The turbidity was monitored by a turbidimeter (2100 P, Hach, CO, USA). The velocity of the water was measured with a handheld Acoustic Doppler Velocimeter (FlowTracker, SonTek, CA, USA). Water samples were filtered through pre-combusted 0.45 μm GF/F filters (Whatman, NJ, USA) and acidified with HCl to analyse the dissolved organic carbon (DOC). The DOC concentrations were determined by an Elementar Liquid-TOC analyser (Frankfurt, Germany). Dissolved silicate (DSi) was estimated with the molybdate blue spectrophotometric method^34^. Total phosphorus (TP) and total nitrogen (TN) were measured colourimetrically after acid hydrolysis with persulfate digestion (30 min, 120 °C). Soluble reactive phosphorus (SRP), nitrite nitrogen (${{NO}}_{2}^{-}$-N), nitrate nitrogen (${{NO}}_{3}^{-}$-N) and ammonium nitrogen (${{NH}}_{4}^{+}$-N) were measured colourimetrically by an AA3 Auto-Analyzer (Seal, Norderstedt, Germany). The detection limits of SRP, ${{NO}}_{2}^{-}$, ${{NO}}_{3}^{-}$ and ${{NH}}_{4}^{+}$ were 0.004, 0.001, 0.003 and 0.003 mg L^−1^, respectively.

### DNA extraction and total bacterial abundance determination

The filters in Section 2.1 were used for DNA extraction by the PowerWater DNA Extraction kit (MoBio, CA, USA) according to the manufacturer’s protocol. The quality and the quantity of DNA were examined by agarose gel (1%) electrophoresis and spectrophotometry (Nano Drop ND 2000, Thermo Scientific, DE, USA).

The extracted DNA was used to determine the 16 S rRNA gene copies of total bacteria in the 14 water samples. The quantitative real-time PCRs were conducted in a 7500 real-time PCR system (Applied Biosystems, Darmstadt, Germany). The details of the PCR amplification and primer sets can be found in the study by Chen^35^. In addition, the PCRs of all samples were performed in triplicate.

### PCR amplification of 16 S rRNA genes and high-throughput sequencing

The detailed operations can be found in the study by Yan^32^. Briefly, the extracted DNA was also used as the template for amplifying the V4 region of 16 S rRNA genes. The PCR primers were validated in the literature and were 515 f (5′-GTGCCAGCMGCCGCGGTAA-3′)/806r (5′-GGACTACHVGGGTWTCTAAT-3′), and PCR amplification was initiated by denaturation at 94 °C for 5 min, followed by 31 cycles at 94 °C for 30 sec, 52 °C for 30 sec, 72 °C for 45 sec, and a final extension step at 72 °C for 10 min. Triplicate PCRs were conducted for each sample to reduce potential PCR bias, then the amplicons were combined equally and gel purified. The obtained DNA library was then sequenced by the Illumina Hiseq. 2000 platform according to the manufacturer’s instructions, which can sequence paired-end reads with length of 100 bp in both forward and reverse directions.

### Data preprocessing

Trimmomatic was applied to the raw sequenced reads to trim the reads with low quality bases. Forward-end reads and reverse-end reads of the raw sequencing data were matched using Flash software and the trim.seqs function in Mothur (version 1.9.1, University of Michigan, Ann Arbor, MI, USA), which can elongate the length of Illumina reads and reduce sequencing error. Reads that contained ambiguous ‘N’ or were shorter than 200 bps were removed. Chimeric and singleton operational taxonomic units (OTUs) were also discarded. Subsequently, quantitative insights into microbial ecology (QIIME, version 1.8.0) was applied to assign the qualified reads to corresponding samples. The QIIME platform was applied to cluster reads into OTUs at 97% similarity. Taxonomy was assigned to the representative sequences by the ribosomal database project (RDP) classifier using the Greengenes Database (version 13_5)^36^.

### Statistical analysis

The Chao1 index, Shannon diversity index and Simpson diversity index were calculated by QIIME. The smallest number of reads among all samples were randomly chosen from each sample for α-diversity analysis to minimize the effect of uneven sampling. A principal component analysis (PCA) was conducted to cluster the sampling sites according to bacterial community composition. A BIOENV analysis was applied to select the environmental variables that could best explain the bacterioplankton variation. Prior to BIOENV analysis, Spearman’s rank correlation was used to remove the highly correlated environmental variables (*r* > 0.80, *p* < 0.05). A Mantel test using the Bray-Curtis matrix of the OTU data and the Euclidean distance matrix of the environmental variables was also applied. A canonical correspondence analysis (CCA) was performed to determine the relationship between the significant environmental variables and the bacterioplankton community structure^25^. A multivariate regression tree (MRT) analysis, which is a constrained clustering method^37^, was conducted using the community data. Clustering may be weak, but if there are strong relationships between the species and the environment, MRT analyses can detect the distinct groups that are not detectable by unconstrained clustering. The OTU data and environmental variables were standardized by z-score transformation. The average value ± standard deviation (SD) of the three replicates were calculated. Significant differences were analyzed using one-way ANOVA followed by Tukey’s multiple comparison test at level of *p* < 0.05. All statistical analyses were performed using the R packages vegan, pheatmap and mvpart (version 3.1.2, R Foundation for Statistical Computing, Vienna, Austria), and SPSS for Windows Version 17.0 (SPSS, Chicago, IL, USA).

### Data availability

All data generated or analysed during this study are included in this article and the Supplementary Information files and are available from the corresponding author on request.

## Results

### Physicochemical characteristics of water samples

The physicochemical characteristics of the surface water in the Yarlung Tsangpo River are presented in Table S1 and Fig. S1 in the Supplementary Information. The elevation of the 14 sampling sites ranged from 2925 m to 4005 m, and the sites were generally considered high plateau areas. The air pressure increased from upstream to downstream and was negatively correlated with elevation (Spearman’s correlation: *r* = −0.935, *p* < 0.001; Supplementary Table S2). The illumination did not significantly change (*p* > 0.05) along the river, while the EC, pH and water temperature at the different sampling sites were comparable. According to Spearman’s correlation test, the velocity and the turbidity were negatively correlated (*r* < 0). More specifically, at the upstream sampling sites (Y.12.R, Y.11.R and Y.10.R), higher velocity co-existed with lower turbidity. However, synchronous changes in velocity and turbidity were observed at the midstream and downstream sites. The ORP and the DO fluctuated and rose, and the concentration of DO peaked downstream of the Zangmu Dam.

The dominant form of dissolved inorganic nitrogen (DIN) was ${{NO}}_{3}^{-}$-N. It increased along the river, peaked in the Zangmu Reservoir, and decreased significantly (*p* < 0.05) downstream of the Zangmu Dam. ${{NO}}_{2}^{-}$-N and ${{NH}}_{4}^{+}$-N were detected at all sites but the concentrations were much lower than that of ${{NO}}_{3}^{-}$-N. The TN pattern was similar to that of ${{NO}}_{3}^{-}$-N at all sampling sites, ranging from 447.02 to 668.95 μg/L. Phosphorus exhibited a different trend than nitrogen, and TP and SRP were closely correlated and generally decreased along the river (Spearman’s correlation: *r* = 0.825, *p* < 0.001). However, TP first significantly increased and then dropped sharply while SRP remained unchanged at the midstream sampling sites (Y.9.R, Y.8.R, Y.7.R, Y.6.L and Y.5.R). The DOC and DSi concentrations ranged from 10.85 to 19.4 mg/L and 9.37 to 10.71 mg/L, respectively. The DOC concentration increased at the upstream sites and decreased further downstream, while a slight increase in DSi concentration was observed downstream of the Zangmu Dam.

### Total bacterial abundance and bacterioplankton diversity along the Yarlung Tsangpo River

**Figure 2:**
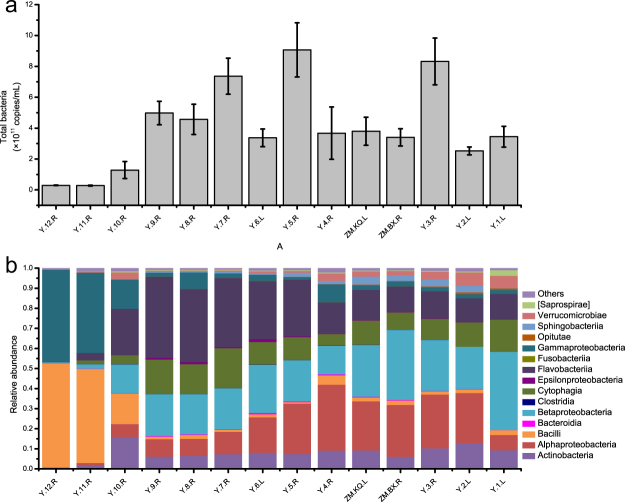
The 16 S rRNA gene copies of total bacteria (**a**) and class-level microbial community profile (**b**) in the water samples collected from 14 sampling sites. The columns with the error bars represent the mean value and standard deviation of triplicates.

The mean number of gene copies of the total bacterial 16 S rRNA from the 14 sampling sites varied from 0.28 × 10^11^ to 9.01 × 10^11^ copies per millilitre in the filtered water samples (Fig. 2a). The upstream sites possessed the lowest total bacterial abundance while the bacteriolplankton biomass measurements at the midstream sites were high. Interestingly, the bacteria biomass remained low in the upstream and downstream areas of the Zangmu Dam (Y.4.R, ZM.KQ.L and ZM.BX.R), suggesting the inhibition of bacterioplankton biomass by the dam.

Overall, the valid sequences obtained in the sampling sites ranged from 65,062 (Y.3.R) to 79,184 (Y.4.R) reads. The sequenced clean reads were assigned to a total of 5022 OTUs after quality filtering. According to the taxonomy results, the number of observed species in the 14 sites varied from 585 to 1740 (Table 1). The number of species increased in the midstream sampling areas and decreased upstream and downstream of the Zangmu Dam. Similar to the pattern of observed species, the Chao1, Shannon and Simpson diversity indices all decreased around the Zangmu Dam and recovered in the downstream sites (Table 1), suggesting that the construction of the dam reduced both the richness and evenness of the bacterioplankton community.

**Table 1:** Measures of α-diversity for samples.

Sites	Observed OTUs	Observed species	Chao1	Shannon	Simpson
Y.12.R	1544^c,d^	1465^c,d^	2503.30^d^	5.68^a^	0.95^a^
Y.11.R	1208^i,j^	1169^i,j^	2318.26^f,g^	3.29^d^	0.74^h^
Y.10.R	629^l^	585^l^	1209.43^i^	2.34^e^	0.68^i^
Y.9.R	1357^h^	1228^h^	2387.68^e,f^	5.25^c^	0.93^c,d^
Y.8.R	1310^j^	1208^h,i^	2289.19^f,g^	5.30^b,c^	0.93^b,c^
Y.7.R	1315^j^	1124^j^	2222.89^g^	5.09^c^	0.92^c,d,e^
Y.6.L	1655^b,c^	1511^b,c^	2743.53^b,c^	5.65^a^	0.93^c,d^
Y.5.R	1574^d,e^	1396^d,e^	2725.02^b,c^	5.26^c^	0.91^f^
Y.4.R	1975^a^	1740^a^	3102.88^a^	5.73^a^	0.91^e,f^
ZM.KQ.L	1455^f^	1369^f^	2688.38^c^	5.28^b,c^	0.91^f^
ZM.BX.R	1382^g^	1299^g^	2443.20^d,e^	5.05^c^	0.89^g^
Y.3.R	1507^d,e^	1450^d,e^	2769.39^b,c^	5.55^a,b^	0.92^e,f^
Y.2.L	1673^b,c^	1532^b,c^	2837.92^b^	5.81^a^	0.94^a,b^
Y.1.L	997^k^	963^k^	1733.35^h^	5.22^c^	0.92^d,e,f^

Chao1 index presents evenness which can measure the relative abundance of the different species. Shannon index and Simpson’s Diversity index are used to measure diversity. The same lowercase letter in one column indicated that the diversity of these sites were not significantly different (*p* > 0.05).

According to the taxonomy results, 15 dominant classes were detected in the bacterioplankton community in the Yarlung Tsangpo River (Fig. 2b), including *Actinobacteria*, *Alphaproteobacteria*, *Bacilli*, *Bacteroidia*, *Betaproteobacteria*, *Clostridia*, *Cytophagia*, *Epsilonproteobacteria*, *Flavobacteriia*, *Fusobacteriia*, *Gammaproteobacteria*, *Opitutae*, *Sphingobacteriia*, *Verrucomicrobiae* and *Saprospirae*. Among the 15 classes that each accounted for more than 1% of the total bacterial abundance, *Alphaproteobacteria*, *Betaproteobacteria* and *Flavobacteriia* were the dominant species in the midstream and downstream sampling sites, and the relative abundances ranged from 0.58% to 32.85%, 0.58% to 39.01% and 0.14% to 40.45%, respectively. Moreover, the relative abundances of *Bacilli* and *Gammaproteobacteria* both exceeded 40% at Y.12.R and Y.11 R, making them the dominant classes of the upstream sites.

### Spatial variation of bacterioplankton along the Yarlung Tsangpo River

**Figure 3:**
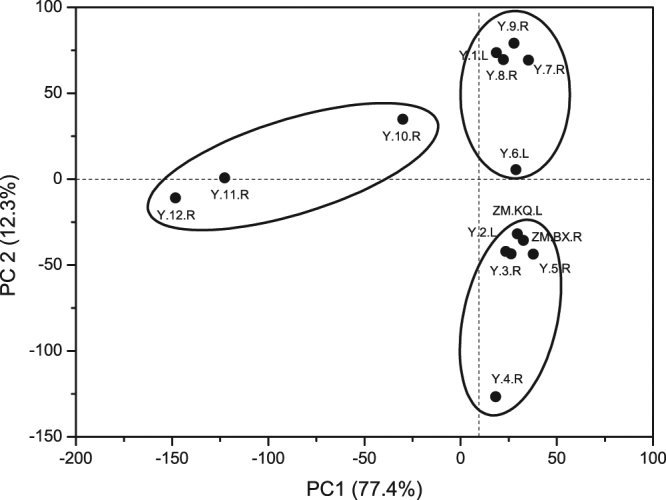
PCA analysis based on 16 S high-throughput sequencing profiles of bacteria communities along the Yarlung Tsangpo River. Each point indicates a sampling site in the river. Distances between any two points on the graph indicate the ecological distance between the community compositions. Numbers within parentheses are the percentage variance explained by each principle component.

PCA was applied in our study to determine the spatial variation of the 14 sampling sites, based on the relative abundances of the bacterioplankton (Fig. 3). The first two principal components, PC1 and PC2, explained 77.4% and 12.3% of the total variance, respectively. The 14 sites were clustered into three groups, as shown in Fig. 3. The first axis of the PCA separated the bacterioplankton communities of the upstream sites (Y.10.R, Y.11.R and Y.12.R) from the other sites, and the second axis generally divided the midstream sites (Y.6.L, Y.7.R, Y.8.R and Y.9.R) from the downstream sites (Y.2.L, Y.3.R, ZM.BX.R, ZM.KQ.L, Y.4.R and Y.5.R). No difference was observed between the dam-associated sites (ZM.BX.R and ZM.KQ.L) and the other downstream sites.

**Figure 4:**
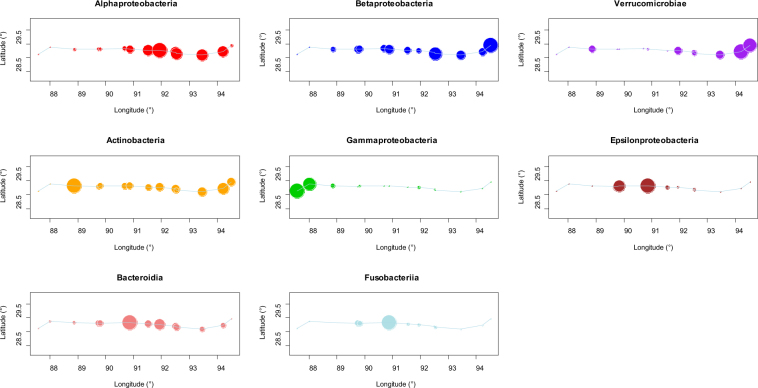
Bubble maps of some class-level bacteria of the Yarlung Tsangpo River. The diameter of bubbles is the ratio between the relative abundance at this site and the maximal relative abundance of all sites. The blue line in figures represents the Yarlung Tsangpo River.

A heat map analysis of the top 30 bacteria at the phylum level is presented in Supplementary Fig. S2 to compare the bacterioplankton composition at the different sampling sites. The Z-scores of the dominant phyla were significantly higher than those of the other phyla at sites of Y.12.R, Y.11.R, Y.10.R and Y.4.R. Interestingly, these sites were located in the upstream portion of the Yarlung Tsangpo River and the Zangmu Reservoir, which indicated the potential effects of location on the bacterioplankton community structure.

Eight typical bacteria classes were selected to analyse the spatial variation along the river. *Alphaproteobacteria*, *Betaproteobacteria* and *Verrucomicrobiae* exhibited comparable spatial variations and their relative abundances increased from upstream to downstream. Conversely, the relative abundances of *Actinobacteria* and *Gammaproteobacteria* decreased from upstream to downstream. Moreover, *Bacteroidia*, *Epsilonproteobacteria* and *Fusobacteriia* reached their highest ratios in the midstream sites of our sampling area.

### Environmental factors influencing bacterioplankton community structure

A BIOENV analysis was conducted to determine the best combination of environmental variables that could explain the bacterioplankton community structure along the Yarlung Tsangpo River. A total of 18 environmental variables were calculated and a combination of 5 variables provided the highest explanation rate (Supplementary Table S3). In general, SRP, ${{NH}}_{4}^{+}$-N, velocity, turbidity and elevation explained 81.70% of the variation in the bacterioplankton community structure and they were selected for the subsequent CCA analysis.

**Figure 5:**
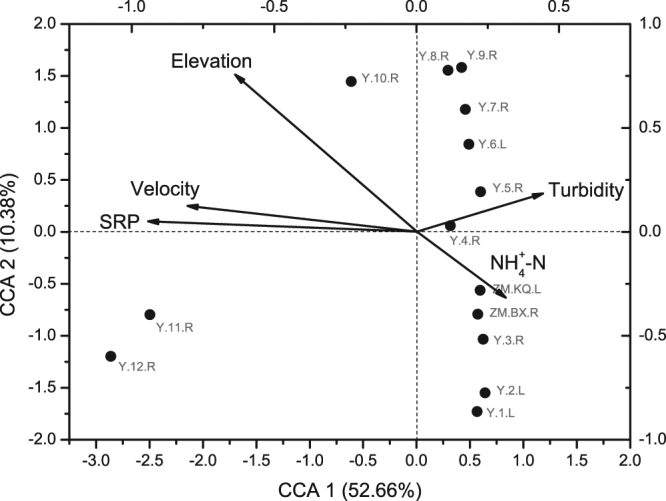
CCA of the bacterioplankton community and the most significant physiochemical variables shaping bacterioplankton community composition and structure. Symbols indicated sampling sites and they were spotted by bottom and left axis. Arrows indicate environmental variables and their relative effects on bacterioplankton community, and they were spotted by top and right axis. The lengths of the arrows indicate how much variance was explained by the corresponding variable. The angles between arrows indicate correlations between individual environmental variables.

According to the CCA analysis, 63.04% of the bacterial community structure variation was explained by CCA1 and CCA2, leaving 36.96% unexplained (Fig. 5). The elevation was positively related to the midstream samples and negatively correlated with the downstream samples. SRP and velocity were both positively correlated with the upstream samples, while they were negatively linked to the midstream and downstream samples. Among the 5 selected environmental variables, SRP, velocity and elevation were the main contributors to the explanation rate. The Mantel test results also confirmed that they were significantly correlated with the bacterial community structure (SRP: r = 0.747, *p* = 0.001; velocity: r = 0.4862, *p* = 0.009; elevation: r = 0.321, *p* = 0.021).

**Figure 6:**
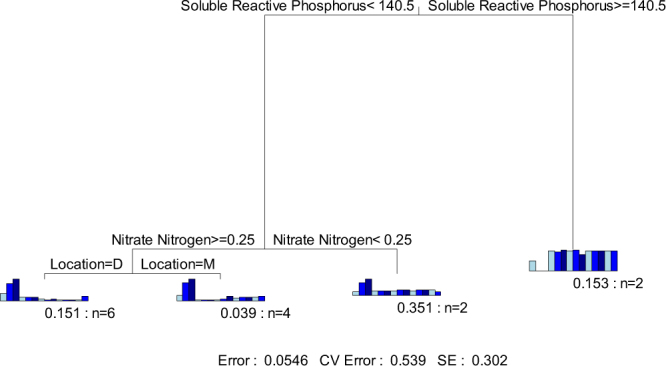
MRT of the bacterioplankton diversity data associated with sampling locations and environmental variables. Each split in the figure is represented graphically as a branch in a tree. Bar plots show the multivariate means of different OTUs at each branch. The numbers of samples within that split are shown under bar plots. D presents downstream and M presents midstream.

An MRT analysis was also applied to determine the influence of environmental variables on the bacterial community structure of the 14 sampling sites (Fig. 6). The MRT was formed by 3 splits and 4 leaves based on the sampling location and physicochemical characteristics of the samples (error = 0.0546, cross-validated relative error = 0.596, standard error = 0.296). The 14 samples were first clustered into 2 groups by SRP concentration and this split explained 88.90% of the variance. Then, the group with SRP concentrations lower than 140.5 μg/L was split by ${{NO}}_{3}^{-}$-N concentration, and this split explained 3.74% of the variance. The 10 samples with ${{NO}}_{3}^{-}$-N concentrations greater than 0.25 mg/L were further separated by sampling location and this split explained 1.90% of the variance. The contributions of the other physicochemical parameters were too small to be recognized in the MRT analysis.

## Discussion

Many researchers have focused on the river ecosystems on the plain, while few studies on plateau rivers have been reported^31,38,39^. Thus, we chose to study a plateau river to supplement the full-scale studies of river ecosystems. Velocity is an important parameter in the river system that can directly indicate the lotic conditions of a river and can quickly be altered by the construction of a dam. According to previous studies, turbidity is significantly associated with velocity^40^. In our study, the velocity and the turbidity changed synchronously in the midstream and downstream sites. However, the velocity downstream of the Zangmu Dam (site ZM.BX.R) increased while the turbidity decreased. Thus, the slow velocity in the Zangmu Reservoir promoted the sedimentation of particulates, which contributed to the interception of upstream particulates by the dam^32^. The reduction of particulates in the water resulted in decreased turbidity downstream of the dam and further led to the asynchrony of velocity and turbidity, which has also been reported in the Jinshajiang River (upper stream of the Yangtze River) and the Lancangjiang River (upper stream of the Mekong River).

Dissolved silicate is an important nutrient for the aquatic ecosystem and was found to slightly decrease in the Zangmu Reservoir and increased after the dam, suggesting the retention of silica by the Zangmu Dam. According to previous studies, decreased silica concentrations are always considered to be induced by river damming and regulation^41,42^.

TN and DIN both increased in the upstream and midstream sites because of the growing anthropogenic activities along the river. The midstream area of the Yarlung Tsangpo River has long been developed for agriculture, and industries and tourism activities have recently and rapidly expanded^29^. These expansions have increased non-point pollution and have led to large nutrient emissions into the main stream of the river. Moreover, the inflow of tributaries, including the Menqu River (between Y.9.R and Y.8.R) and the Lhasa River (between Y.7.R and Y.6.L), have further contributed to the nutrient increases in the main stream, as intensive urbanization and tourism have also occurred along these tributaries. TN and DIN also decreased at some sites (Y.6.L, Y.5.R and Y.4.R), which was due to the particulate precipitation resulting from the flat landscape features of this area. The concentrations of TN and DIN decreased after the Zangmu Reservoir, which was associated with dilution by the tributaries and the biogeochemical processes occurring in the reservoir^27^.

Opposite from the nitrogen pattern, TP and SRP decreased along the Yarlung Tsangpo River, and this decrease is linked to the velocity conditions (confirmed by Spearman’s rank correlation test). As the velocity at the upstream sites was relatively high, the release of phosphorus from the sediment to the water was enhanced^43,44^, resulting in the high concentrations in upstream areas. At the midstream sites, TP exhibited a sharp increase while the SRP concentration remained constant, indicating that the majority of the phosphorus emission were in the organic form. The increase in organic phosphorus was related to the intense agricultural activities and the increasing urban discharge in this area, as organic phosphorus was commonly used in anthropogenic activities^45,46^. Interestingly, the TP concentration was lower at some midstream sites (Y.6.L, Y.5.R and Y.4.R), which was related to sedimentation over the flat landscape and decreased input by reduced inflow of tributaries in this area. Similar to the nitrogen pattern, both TP and SRP were retained by the Zangmu Dam, which has also been observed in previous studies^47,48^.

Consequently, the concentrations of nutrients in the water corresponded to the economic development gradient and the river regulation conditions from the upstream to the downstream along the Yarlung Tsangpo River.

In our study, the total bacteria abundance and the α-diversity were both reduced around the Zangmu Dam, indicating that river damming reduced the biomass and diversity of bacterioplankton. The construction of dams disturbs river continuum^49^. Moreover, the impoundment of dams would convert main streams into reservoirs with slow-moving water, and many corresponding hydrological parameters, such as velocity and turbidity, would change. As bacterioplankton biomass and diversity can respond quickly to changing environmental conditions^14^, it is not surprising that the decreased biomass and diversity of bacterioplankton were observed in our study and the study by Lu and colleagues^50^. Interestingly, no abrupt change in bacterioplankton community structure was observed in the Zangmu Reservoir according to the PCA, which differs from the response of biomass and diversity to the construction of dam. The discrepancy of dam effect on biomass, diversity and community structure of bacterioplankton was related with the running time and the reservoir capacity of the Zangmu Dam. Since the dam began to operate in late 2015, the time for water storage was short and its effect on river continuum was not noticeable. Moreover, the capacity of the Zangmu Reservoir is not large (8.66 × 10^7^ m^3^) when compared with that of the Three Gorges Reservoir (3.93 × 10^10^ m^3^)^32^, thus the retention of nutrients by the Zangmu Reservoir was not remarkable. According to previous studies, hydrological condition^20^ and nutrient distribution^51^ can shape the community structure of bacterioplankton in rivers, as the adaptive bacteria are sorted in the altered environment and dominate the community. As a result, little alteration of community structure was observed between upstream and downstream sites of the Zangmu Dam. Actually, the very few existing studies which compared the bacterioplankton communities upstream and downstream of the dams presented contrasting results. Significant difference of bacterioplankton community composition was observed upstream and downstream of the dam at the small Sinnamary River^52^, while the large impoundments in the Danube River caused undiscerned effects on bacterioplankton communities^53^. Consequently, there still remains a lack of knowledge concerning the effect of river damming on bacterioplankton community composition and in-depth studies are needed urgently.

According to our taxa results, the bacterioplankton community in the Yarlung Tsangpo River was dominated by *Proteobacteria*, *Bacteroidetes*, *Actinobacteria*, *Firmicutes*, *Verrucomicrobia* and *Fusobacteria*, which are usually observed in freshwater and are important contributors to biogeochemical processes^54,55^. For example, *Alphaproteobacteria*, *Betaproteobacteria* and *Gammaproteobacteria* were reported to be involved in the balance of the global nitrogen budget through nitrogen fixation or denitrification^14^. The upstream sites were dominated by *Firmicutes*, including *Bacilli* and *Clostridia*, which can survive in extreme environments^56^ such as the low temperature or the intense light irradiation in the Tibetan Plateau. Different from the upstream sites, the midstream sites were dominated by *Bacteroidetes*, which are believed to degrade high-molecular-weight organic compounds^57^. Agricultural activities have long been developed in the midstream area, and the expansion of urbanization and tourism has increased the anthropogenic disturbances in recent years, leading to an increasing input of organic-rich wastewater into the Yarlung Tsangpo River and the promotion of *Bacteroidetes* growth. *Proteobacteria* often dominate the bacterial community in lakes and rivers on the plain^58,59^, and they were also the dominant phylum in the downstream sites of the Yarlung Tsangpo River, indicating the similarity between the downstream areas of the plateau river and plain freshwater systems. As a result, the bacterioplankton community composition presented endemic patterns and can be attributed to anthropogenic disturbances along the Yarlung Tsangpo River.

Bacterioplankton are generally passive dispersers that are in states of flux at individual sites with constant immigration occurring from upstream areas and emigration occurring to downstream areas following the water flows^19^. In general, the distribution of bacterioplankton community changed gradually along the Yarlung Tsangpo River following the dispersal dynamics. However, it was observed that the dominant bacteria suddenly changed from *Firmicutes* to *Bacteroidetes* in upstream and midstream sites and the bacterioplankton diversity fluctuated in dam-associated sites, which could be attributed to anthropogenic disturbances. In consequence, the directional dispersal dynamics of bacterioplankton in rivers can be affected by anthropogenic activities which should be taken into account to protect river ecosystem in the future.

Bacterioplankton communities are highly diverse in natural aquatic ecosystems, and they can experience shifts in composition in response to spatial environmental gradients^60–62^. Fluctuations of environmental variables may result in changes in the functional roles of the bacterial communities in the biogeochemical nutrient cycles^63–65^. Unified results from previous studies do not exist, therefore, 18 environmental variables were measured to determine the critical factors that influenced the bacterioplankton community in our study. SRP, ${{NH}}_{4}^{+}$-N, velocity, turbidity and elevation were the main environmental factors influencing the bacterioplankton community along the Yarlung Tsangpo River. SRP and ${{NH}}_{4}^{+}$-N both serve as the available essential nutrients for bacterial growth and the trophic types have been proven to have profound effects on bacterial communities^66,67^. In the midstream of the Yarlung Tsangpo River, increasing amount of nutrient was loaded into the water due to the anthropogenic activities in this area. The dominant phylum of bacterioplankton community was *Bacteroidetes*, which is considered to degrade high-molecular-weight organic compounds^57^. The relationship between the increased nutrients and the dominance of *Bacteroidetes* was also observed in previous studies^25,57^ and the fluctuation of nutrient was also confirmed to shape bacterial community in the Dongjiang River^14^ and in the Lake Bourget^63^.

Velocity and turbidity, which can be easily altered by topographic features of the river and dam construction, were closely correlated. Lower velocity can result in particle sedimentation in the water column, decreasing the turbidity in rivers. The changes of particle content in water will greatly influence the ratio of bacteria since the bacterioplankton are composed of free-living and particle-associated bacterial^20^. Similar to our results, in the Danube River, turbidity was also considered to shape the bacterioplankton community through its link with particle content^31^. In consequence, velocity and turbidity can contribute to the bacterioplankton community in the Yarlung Tsangpo River.

In our study, elevation was another important factor governing the bacterioplankton community in the river. According to previous studies, the change of elevation often co-occurred with varied climatic parameters, including water temperature and light, which play important roles in various physiological activities in different bacteria^58,62^. In the Yarlung Tsangpo River, the dominant bacteria in upstream sites was *Firmicutes*, which can survive in extreme environments^56^ like the low temperature or the intensive light irradiation in the high-elevation sites, further supporting the contribution of elevation on bacterioplankton community in the river.

Aside from physicochemical environmental variables, some biological factors have also been considered to be important in plain rivers. Phytoplankton may compete with bacterioplankton for nutrients, and predation by protists may impede the growth of bacterioplankton; thus, phytoplankton and protistan bacterivory may also be associated with bacterioplankton community dynamics^68–70^. The chlorophyll-*a* concentration was monitored along the Yarlung Tsangpo River, but it was under the detection limit in all sampling sites. It may be due to that the cumulative impact of anthropogenic activities was insufficient to support the growth of phytoplankton in the river. As a result, we focused on the abiotic variables rather than the biotic factors, which was one of the limitations in our present study. But in the future, as the development of urbanization and tourism will be enhanced and more hydropower stations will be constructed, the growth of phytoplankton may be promoted. So, more biotic factors will be included in the next sampling to improve our understanding of factors shaping the bacterioplankton community in the Yarlung Tsangpo River.

The sampling in this study was conducted over 940 km; therefore, our results were consistent with the hypothesis proposed by Ge that environmental factors are dominant drivers at the local scale (<1000 km) while historical events control the bacterial community at regional scales (>1000 km)^71^. Many researchers have advised that bacteria community shifts could be sensitive indicators of environmental changes in aquatic systems. However, environmental variables that commonly govern bacterioplankton communities have not yet been identified, especially in rivers, due to the lack of information. Consequently, more observational and experimental data are needed to reveal the hierarchical environmental controls on river bacterioplankton communities.

## Conclusion

In summary, our results suggest that nutrient variations correspond to anthropogenic activities, and river damming has an important influence on biogeochemical nutrient cycling, bacterioplankton biomass and community diversity in the Yarlung Tsangpo River. However, significant alterations of the bacterioplankton community structure were not observed between upstream and downstream of the dam. In addition, the spatial distribution of the bacterioplankton community was linked with anthropogenic activities and changed gradually along the river, and the bacterioplankton composition had an endemic pattern on the Tibetan Plateau. Moreover, SRP, elevation, ${{NH}}_{4}^{+}$-N, velocity and turbidity were the main environmental factors that shaped the bacterioplankton community in the river. Our study offers the first insights into the diversity and composition of bacterioplankton in a dammed river in a plateau region. In the future, more studies are needed to investigate how the bacterioplankton community responds to anthropogenic activities along rivers, especially along plateau rivers.

## Electronic supplementary material

Supplementary Information
