# Supplementary material for: Shift in bacterioplankton diversity and structure: Influence of anthropogenic disturbances along the Yarlung Tsangpo River on the Tibetan Plateau, China
Source: Sci Rep. 2017 Oct 2;7:12529. doi: 10.1038/s41598-017-12893-4 (PMC5624883; doi:10.1038/s41598-017-12893-4)
Supplement: Supplementary file 1 — Supplementary Information [file 41598_2017_12893_MOESM1_ESM.pdf]

# Supplementary Information

# Shift in bacterioplankton diversity and structure: Influence of anthropogenic disturbances along the Yarlung Tsangpo River on the Tibetan Plateau, China

Peifang Wang[a]*, Xun Wang[a]*, Chao Wang[a], Lingzhan Miao[a], Jun Hou[a], Qiusheng Yuan[a]

[a] Key Laboratory of Integrated Regulation and Resource Development on Shallow Lakes, Ministry of Education, College of Environment, Hohai University, Nanjing, 210098, China.

* Corresponding author. Address: College of Environment, Hohai University, 1 Xikang Road, Nanjing, 210098, China.

Tel.: +86-25-83786028; Fax: +86-25-83787332.

E-mail: pfwang2005@hhu.edu.cn (P. Wang), xwang2014@hhu.edu.cn (X. Wang).

**Table S1** Details of the samples including geographic location and physical characteristics.

| Sites | Longitude | Latitude | Elevation (m) | Illumination (kWh/m$^2$/day) | Air Pressure (kPa) | Velocity (m/s) | Turbidity (NTU) | EC (μs/cm) | ORP (mV) | DO (mg/L) | pH | T (℃) |
|---|---|---|---|---|---|---|---|---|---|---|---|---|
| Y.12.R | E 87° 34.556′ | N 29° 07.293′ | 4005 | 5.61 | 56.91 | 2±0.11 | 24.8±0.24 | 338±12.34 | 130.4±2.50 | 7.63±0.07 | 8.17±0.10 | 8.4±0.15 |
| Y.11.R | E 88° 00.478′ | N 29° 22.721′ | 3946 | 5.61 | 56.77 | 3±0.14 | 19.3±0.20 | 275±17.06 | 119.8±1.78 | 7.72±0.06 | 8.45±0.11 | 6.4±0.13 |
| Y.10.R | E 88° 51.656′ | N 29° 19.147′ | 3847 | 5.64 | 56.22 | 2±0.12 | 20.9±0.06 | 266±17.04 | 124.8±1.14 | 7.42±0.07 | 8.74±0.11 | 8.9±0.12 |
| Y.9.R | E 89° 45.164′ | N 29° 17.994′ | 3776 | 5.69 | 56.91 | 0.5±0.01 | 33.5±0.15 | 264±16.31 | 105.9±0.85 | 7.58±0.05 | 9.2±0.15 | 8.7±0.13 |
| Y.8.R | E 89° 49.052′ | N 29° 18.396′ | 3783 | 5.69 | 56.83 | 2±0.11 | 34.4±0.12 | 255±17.50 | 132.7±0.70 | 8.28±0.07 | 8.27±0.12 | 8.4±0.08 |
| Y.7.R | E 90° 41.344′ | N 29° 19.541′ | 3604 | 5.71 | 57.35 | 1.2±0.08 | 27.9±0.09 | 261±16.88 | 113.1±0.71 | 8.03±0.06 | 8.23±0.12 | 8.5±0.09 |
| Y.6.L | E 90° 52.146′ | N 29° 18.829′ | 3575 | 5.71 | 57.28 | 0.5±0.02 | 27.4±0.08 | 240±13.08 | 126.1±1.05 | 7.37±0.05 | 8.25±0.13 | 8±0.15 |
| Y.5.R | E 91° 32.292′ | N 29° 16.196′ | 3585 | 5.72 | 58.24 | 0.6±0.01 | 59.7±0.25 | 234±12.86 | 147.4±0.42 | 7.24±0.05 | 8.17±0.03 | 9.7±0.15 |
| Y.4.R | E 91° 56.415′ | N 29° 15.946′ | 3561 | 5.72 | 58.15 | 1±0.09 | 64.3±0.15 | 240±12.94 | 133±0.67 | 6.98±0.06 | 7.79±0.07 | 8.8±0.11 |
| ZM.KQ.L | E 92° 30.827′ | N 29° 11.515′ | 3279 | 5.76 | 58.74 | 0.2±0.01 | 39.4±0.21 | 237±12.91 | 121.6±0.50 | 8.61±0.07 | 8.06±0.12 | 9.9±0.12 |
| ZM.BX.R | E 92° 31.835′ | N 29° 09.142′ | 3273 | 5.76 | 58.69 | 1.5±0.13 | 36.3±0.18 | 237±11.87 | 143.4±0.51 | 9.19±0.06 | 7.63±0.06 | 9.9±0.13 |
| Y.3.R | E 93° 27.151′ | N 29° 06.292′ | 3075 | 5.62 | 61.67 | 1.2±0.11 | 45±0.17 | 232±10.94 | 147.4±0.47 | 7.83±0.07 | 7.96±0.08 | 9.7±0.09 |
| Y.2.L | E 94° 12.929′ | N 29° 13.695′ | 2933 | 5.53 | 65.44 | 0.33±0.02 | 34.9±0.11 | 287±20.11 | 161.4±0.21 | 7.72±0.06 | 7.16±0.11 | 9.4±0.11 |
| Y.1.L | E 94° 31.436′ | N 29° 27.101′ | 2925 | 5.57 | 65.82 | 0.2±0.02 | 6.17±0.02 | 170±13.58 | 140.8±0.43 | 8.97±0.07 | 8.83±0.13 | 17.2±0.15 |

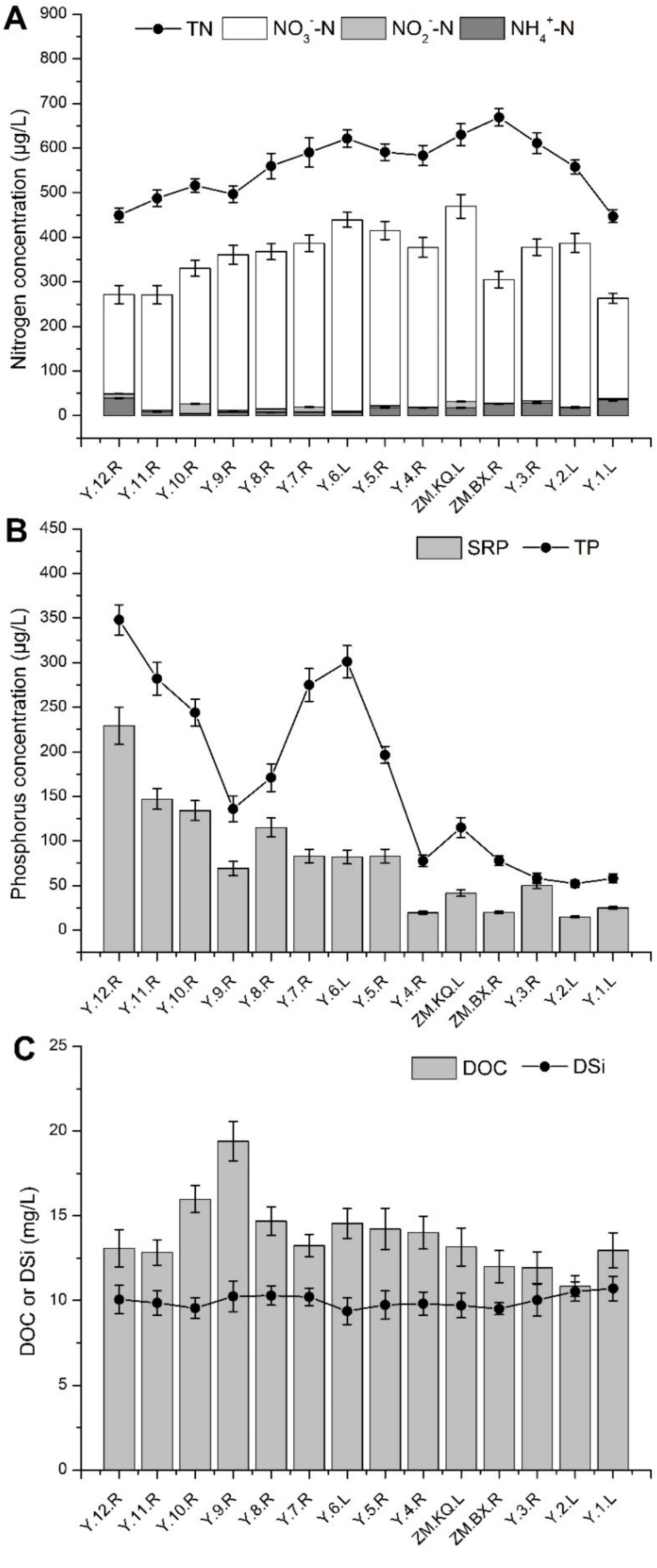


**Figure S1** Main nutrient characteristics of surface water samples in the Yarlung Tsangpo River.

**Table S2** Results of the Spearman's rank (*r*) correlation analysis and the difference between two environmental variables (*, $p$<0.05; **, $p$<0.01).

In our study, the correlation between two environmental variables were considered close when $r$>0.8 or $r$<-0.8, and $p$<0.05.

| | DSi | SRP | TP | TN | $NO_2^-$-N | $NH_4^+$-N | $NO_3^-$-N | Velocity | Turbidity | Elevation | T | EC | DOC | ORP | pH | DO | Illumination | Air Pressure |
|---|---|---|---|---|---|---|---|---|---|---|---|---|---|---|---|---|---|---|
| **DSi** | 1.000 | -0.077 | -0.315 | -0.335 | 0.086 | -0.169 | -0.211 | -0.139 | -0.248 | -0.174 | 0.126 | 0.088 | -0.178 | 0.040 | 0.359 | 0.231 | -0.588* | 0.125 |
| **SRP** | | 1.000 | 0.825** | -0.374 | 0.654* | -0.450 | -0.220 | 0.690** | -0.525 | 0.877** | -0.606* | 0.421 | 0.385 | -0.524 | 0.590* | -0.205 | -0.161 | -0.827** |
| **TP** | | | 1.000 | -0.033 | 0.547* | -0.603* | 0.035 | 0.482 | -0.484 | 0.812** | -0.659* | 0.461 | 0.572 * | -0.680** | 0.502 | -0.267 | 0.126 | -0.835** |
| **TN** | | | | 1.000 | -0.173 | -0.121 | 0.378 | -0.244 | 0.410 | -0.185 | 0.000 | -0.088 | 0.119 | -0.255 | -0.279 | 0.166 | 0.529 | 0.106 |
| **$NO_2^-$-N** | | | | | 1.000 | -0.265 | 0.075 | 0.196 | -0.209 | 0.467 | -0.042 | 0.139 | 0.256 | -0.426 | 0.345 | 0.111 | 0.009 | -0.344 |
| **$NH_4^+$-N** | | | | | | 1.000 | -0.387 | -0.171 | 0.184 | -0.452 | 0.497 | -0.339 | -0.299 | 0.584* | -0.469 | 0.312 | -0.177 | 0.651* |
| **$NO_3^-$-N** | | | | | | | 1.000 | -0.499 | 0.554* | -0.180 | -0.015 | -0.135 | 0.176 | -0.029 | -0.317 | -0.279 | 0.543* | 0.159 |
| **Velocity** | | | | | | | | 1.000 | -0.250 | 0.708** | -0.538* | 0.436 | 0.049 | -0.163 | 0.124 | -0.060 | -0.132 | -0.712** |
| **Turbidity** | | | | | | | | | 1.000 | -0.371 | 0.355 | -0.385 | -0.051 | 0.453 | -0.708** | -0.167 | 0.590* | 0.396 |
| **Elevation** | | | | | | | | | | 1.000 | -0.750** | 0.650* | 0.587* | -0.612* | 0.464 | -0.365 | -0.044 | -0.935** |
| **T** | | | | | | | | | | | 1.000 | -0.667** | -0.481 | 0.508 | -0.313 | 0.417 | 0.187 | 0.772** |
| **EC** | | | | | | | | | | | | 1.000 | 0.192 | -0.384 | 0.099 | -0.282 | -0.410 | -0.609* |
| **DOC** | | | | | | | | | | | | | 1.000 | -0.469 | 0.436 | -0.418 | 0.256 | -0.519 |
| **ORP** | | | | | | | | | | | | | | 1.000 | -0.620* | 0.051 | -0.145 | 0.634* |
| **pH** | | | | | | | | | | | | | | | 1.000 | -0.064 | -0.274 | -0.532 |
| **DO** | | | | | | | | | | | | | | | | 1.000 | -0.039 | 0.385 |
| **Illumination** | | | | | | | | | | | | | | | | | 1.000 | 0.004 |
| **Air Pressure** | | | | | | | | | | | | | | | | | | 1.000 |

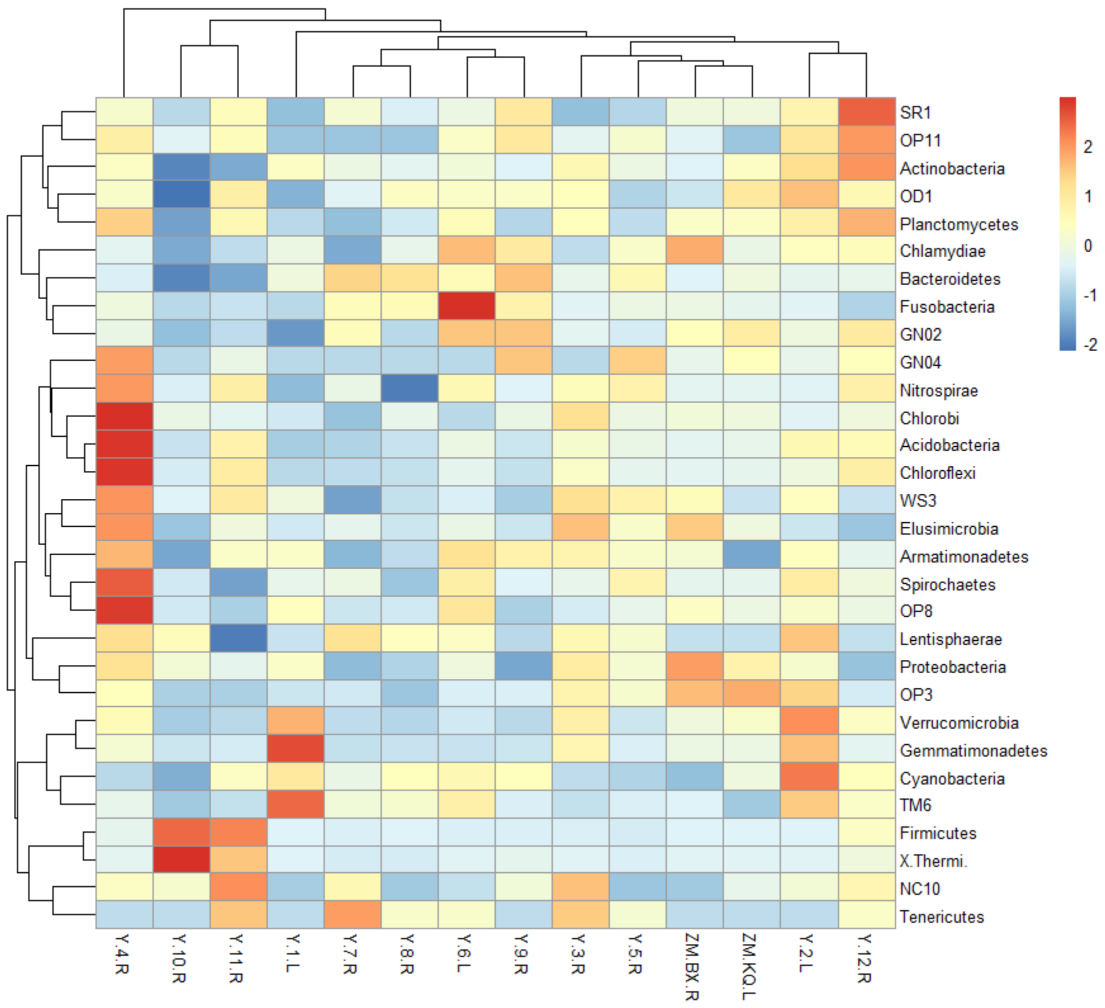


**Figure S2** Heat map analysis of the 30 most abundant bacteria at the phylum level. The relative abundance of each OTU was normalized across samples, converted into a Z-score for its occurrence in each sample. Dendrograms based on the Bray-Curtis similarity measure are also shown.

**Table S3** Results of the BIOENV analysis defining a subset of environmental variables that best explains the community structure. A series of a single or a combination of environmental variables is presented. Eighteen environmental variables were calculated. Value in bold indicated the highest explanation of the variance.

| Size | Variables | Correlation |
|---|---|---|
| 1 | SRP | 0.6957 |
| 2 | SRP, EC | 0.7422 |
| 3 | SRP, $NH_4^+$-N, Velocity | 0.7743 |
| 4 | SRP, $NH_4^+$-N, Velocity, Turbidity | 0.7969 |
| 5 | **SRP, $NH_4^+$-N, Velocity, Turbidity, Elevation** | **0.8170** |
| 6 | SRP, $NO_2^-$-N, $NH_4^+$-N, Velocity, Turbidity, Elevation | 0.8081 |
| 7 | SRP, $NO_2^-$-N, $NH_4^+$-N, Velocity, Turbidity, Elevation, EC | 0.7860 |
| 8 | SRP, $NO_2^-$-N, $NH_4^+$-N, Velocity, Turbidity, Elevation, EC, pH | 0.7609 |
| 9 | SRP, TP, $NO_2^-$-N, $NH_4^+$-N, Velocity, Turbidity, Elevation, EC, pH | 0.7513 |
| 10 | SRP, TP, $NO_2^-$-N, $NH_4^+$-N, $NO_3^-$-N, Velocity, Turbidity, Elevation, EC, DOC | 0.7247 |
| 11 | SRP, TP, $NO_2^-$-N, $NH_4^+$-N, $NO_3^-$-N, Velocity, Turbidity, Elevation, T, EC, pH | 0.7027 |
| 12 | SRP, TP, TN, $NO_2^-$-N, $NH_4^+$-N, $NO_3^-$-N, Velocity, Turbidity, Elevation, T, EC, pH | 0.6931 |
| 13 | SRP, TP, TN, $NO_2^-$-N, $NH_4^+$-N, $NO_3^-$-N, Velocity, Turbidity, Elevation, T, EC, pH, DO | 0.6758 |
| 14 | DSi, SRP, TP, TN, $NO_2^-$-N, $NH_4^+$-N, $NO_3^-$-N, Velocity, Turbidity, Elevation, T, EC, pH, DO | 0.6443 |
| 15 | DSi, SRP, TP, TN, $NO_2^-$-N, $NH_4^+$-N, $NO_3^-$-N, Velocity, Turbidity, Elevation, T, EC, DOC, DO, Air Pressure | 0.6079 |
| 16 | DSi, SRP, TP, TN, $NO_2^-$-N, $NH_4^+$-N, $NO_3^-$-N, Velocity, Turbidity, Elevation, T, EC, pH, DO, Illumination, Air Pressure | 0.5758 |
| 17 | DSi, SRP, TP, TN, $NO_2^-$-N, $NH_4^+$-N, $NO_3^-$-N, Velocity, Turbidity, Elevation, T, EC, DOC, pH, DO, Illumination, Air Pressure | 0.5490 |
| 18 | DSi, SRP, TP, TN, $NO_2^-$-N, $NH_4^+$-N, $NO_3^-$-N, Velocity, Turbidity, Elevation, T, EC, DOC, ORP, pH, DO, Illumination, Air Pressure | 0.5155 |
